# Folic acid supplementation improves cognitive function by reducing the levels of peripheral inflammatory cytokines in elderly Chinese subjects with MCI

**DOI:** 10.1038/srep37486

**Published:** 2016-11-23

**Authors:** Fei Ma, Tianfeng Wu, Jiangang Zhao, Aili Song, Huan Liu, Weili Xu, Guowei Huang

**Affiliations:** 1 Department of Epidemiology and Biostatistics, School of Public Health, Tianjin Medical University, Tianjin, China; 2 Department of Nutrition and Food Science, School of Public Health, Tianjin Medical University, Tianjin, China; 3 Community Health Service Center, Sanhuailu Street, Binhai New District, Tianjin, China; 4 Aging Research Center, Department Neurobiology, Care Sciences and Society, Karolinska Institutet, Stockholm, Sweden

## Abstract

This study aimed to evaluate whether folic acid supplementation would improve cognitive performance by reducing serum inflammatory cytokine concentrations. This RCT was performed in Tianjin, China. Participants with mild cognitive impairment (MCI) were randomly assigned to the folic acid (400 μg/day) or conventional treatment groups. Neuropsychological tests were administered, and folate, homocysteine, vitamin B_12_, IL-6, TNF-α, Aβ-42, and Aβ-40 were measured at baseline and at 6- and 12-month time points.152 participants (folic acid: 77, conventional: 75) completed the trial. Significant improvements in folate (ηp^2^ = 0.703, P = 0.011), homocysteine (ηp^2^ = 0.644, P = 0.009), Aβ-42 (ηp^2^ = 0.687, P = 0.013), peripheral IL-6 (ηp^2^ = 0.477, P = 0.025), TNF-α (ηp^2^ = 0.709, P = 0.009) levels were observed in folic acid group compared with conventional group. Folic acid supplementation improved the Full Scale Intelligence Quotient (P = 0.028; effect size d = 0.153), Information (P = 0.031; d = 0.157) and Digit Span (P = 0.009; d = 0.172) scores at 12 months compared with conventional treatment. Based on these findings, daily oral administration of a 400-μg folic acid supplement to MCI subjects for 12 months can significantly improve cognitive performance and reduce peripheral inflammatory cytokine levels.

Currently, dementia is a substantial public health concern due to the exponentially increasing number of older adults. Developing novel strategies to protect cognition in the elderly population is critical for managing the disease burden and cost of care^1^. The pathogenesis and progression of Alzheimer’s disease (AD) (the first cause of dementia) are associated with various inflammatory processes^2^,^3^. Indeed, inflammatory processes play at least some role in the pathology of AD and mild cognitive impairment (MCI)^4^, which represents an intermediate state between normal cognitive aging and dementia. Particularly intriguing are peripheral inflammatory cytokines, as increased levels of IL-1β, IL-6, and TNF-α have been observed in both the peripheral blood^5^,^6^ and autopsy specimens^7^ of patients with mild to moderate late-onset AD. Current treatments for AD and other dementias are limited; therefore, effective nutritional intervention approaches for improving cognitive deficits that reduce the peripheral inflammatory cytokine levels have garnered special attention.

Folate is the generic term for this water-soluble B-complex vitamin. Folate, vitamin B_12_ and vitamin B_6_ support the metabolic availability of methyl groups and thus facilitate the remethylation of homocysteine to methionine. Serum homocysteine levels are associated with both cognitive decline and dementia. Based on the known effects of folate and vitamin B_12_ deficiencies and abnormal homocysteine metabolism on the development and maintenance of the nervous system, several plausible mechanisms through which high homocysteine levels might increase the risk of dementia have been postulated (e.g., an impact on cerebrovascular pathology, direct neurotoxic effects or an influence on the neurofibrillary tangle burden and amyloid-β accumulation through impacts on methylation reactions)^8^,^9^,^10^,^11^,^12^,^13^,^14^. However, none of these mechanisms have been proven unequivocally. Specifically, it is unknown whether the associations are causal^15^,^16^. Resolving the uncertainty is of practical importance because folic acid supplementation reduces the serum homocysteine levels and is therefore a possible method to help prevent dementia or age-related cognitive decline. Randomized trials studying the effects of folic acid treatments on preventing cognitive decline have been performed, but the results are not conclusive^17^,^18^,^19^. In epidemiological studies, low folate concentrations were shown to induce an inflammatory state that may explain the relationship between vitamin B deficiency and the risk of AD^20^.

In China, the prevalence of folate deficiency is greater than 20%. The level of folate intake is usually 30–40% less than the recommended dietary allowance, due to a lack of folic acid fortification programs and the use of traditional cooking methods that may cause folate to be lost from vegetables^21^. The effects of folic acid supplementation on cognitive performance might be greater in countries such as the United States that have mandated folic acid fortification in flour than they would be in populations without mandatory fortification^19^,^22^. Thus, evidence from large trials in populations without grain fortification is needed. We have previously reported a beneficial effect of six-month folic acid supplementation on cognitive function in people with MCI^23^. In the present study, we aimed to: 1) examine the association of twelve-month folic acid supplementation with changes in cognitive performance over time using the longitudinal data from this trial, and 2) explore the potential role of peripheral inflammatory cytokines in this association.

## Results

### Participants’ baseline characteristics

**Figure 1:**
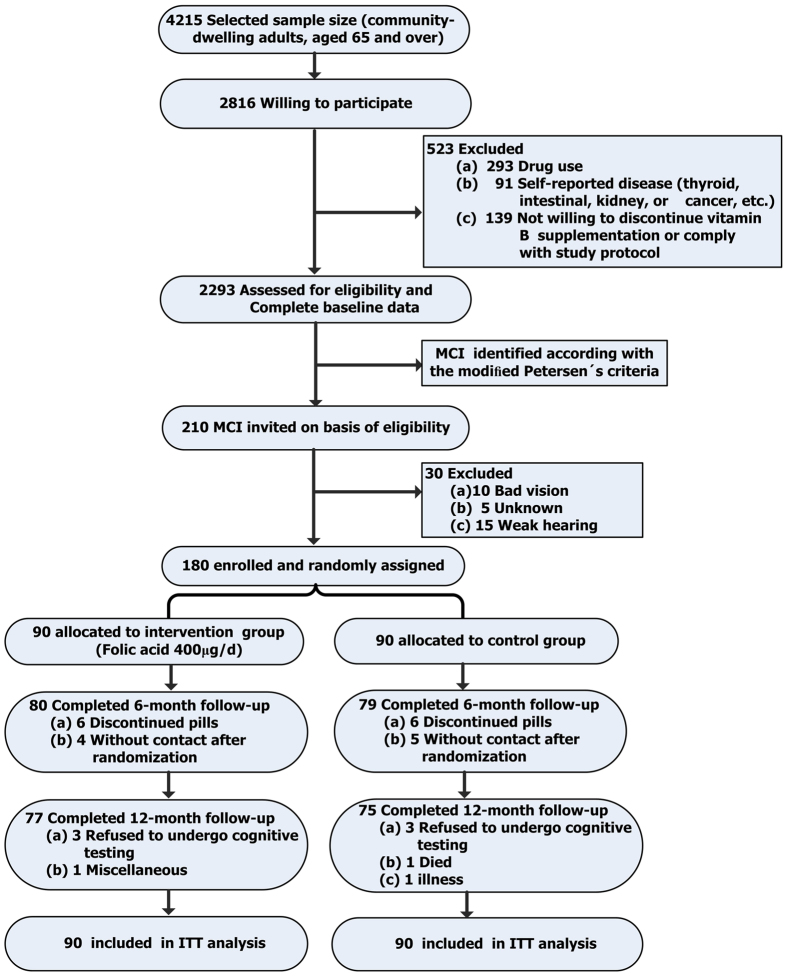
Flow diagram of the subjects’ enrollment from the initial contact through study completion.

Using random cluster sampling, six geographically convenient communities with a high proportion of older residents who were all native Chinese speakers were selected from the Binhai New District, Tianjin, China. Of the 4215 individuals selected, 2816 (66.8%) agreed to participate, but only 2293 participants were eligible for the clinical, physical, and neuropsychological examinations. Two hundred ten subjects with MCI were selected from these individuals using previously determined criteria for MCI (Fig. 1). Of the 210 MCI individuals screened, 168 met the inclusion criteria and were assigned randomly to the folic acid supplementation or conventional-treatment groups. Dropout rates were 8.33% (7/84) in the folic acid group and 10.7% (9/84) in the conventional-treatment group. There was no significant difference in dropout rates between the two groups (*χ*^*2*^ = 0.276, P = 0.834). Baseline characteristics of the study population are shown in Table 1.

### Levels of peripheral inflammatory cytokines and biomarkers of folate status

Repeated-measures ANOVAs of the peripheral inflammatory cytokine levels during the twelve-month period showed greater increases in the serum folic acid levels (+34.66%) in the participants in the folic acid treatment group than in the participants in the conventional-treatment group (+1.55%). Serum homocysteine (Hcy) levels showed a greater decrease in the intervention group (−39.85%) compared to the conventional-treatment group (−7.05%). The serum IL-6 percentages showed substantial decreases in both groups (P = 0.025, *ηp*^2^ = 0.477), but these decreases were greater in the folic acid intervention group (−18.67%) than in the conventional-treatment group (−5.75%); similarly, the serum TNF-α percentages showed substantial decreases in both groups (P = 0.009, *ηp*^2^ = 0.709) but were greater in the intervention group (−26.96%) than in the conventional-treatment group (−0.32%). Additionally, the serum Aβ−42 levels decreased in both groups at month 12, but this decrease was greater in the intervention group (−7.37%) than in the conventional-treatment group (−1.98%). However, no significant differences in the vitamin B_12_ or Aβ-40 levels were observed (Table 2).

### Cognitive status

The repeated-measures analyses of covariance (ANCOVA) revealed significant interaction effects for the Full Scale IQ, Information, and Digit Span tests over the twelve-month period (Table 3). The results of the other neuropsychological tests were not significant. The intervention group showed a marginally significant improvement in the Full Scale IQ over the twelve-month period compared to the conventional-treatment group (P = 0.044, *ηp*^2^ = 0.082). Based on the analysis of each domain, the Digit Span scores showed marked increases in both groups at month 12. The Information scores for both groups increased at month 12 but were greater in the intervention group than in the conventional-treatment group.

In our study, the median plasma vitamin B_12_ concentration was 569.24 pg/mL. We classified all subjects into two groups to adjust for confounding effects of the plasma vitamin B_12_ level: low vitamin B_12_ status (<569.24 pg/mL) and high vitamin B_12_ status (≥569.24 pg/mL). Then, we evaluated hypotheses concerning the differences in the changes observed between the folic acid supplementation group and the control group using mixed-model repeated-measures ANOVAs in each group. The data displayed in Table 4 summarize the interaction between the serum folate levels and vitamin B_12_ status in relation to cognitive impairment. In the group with a high vitamin B_12_ status, the intervention group showed a marginally significant improvement in the Full Scale IQ over the twelve-month period compared to the conventional-treatment group (P = 0.032, *ηp*^2^ = 0.109). There were no significant findings in the group with low vitamin B_12_ status.

### Mixed-model analysis of the association between cognitive improvement and the baseline levels of laboratory variables

Mixed-model repeated-measures ANOVAs were used to determine particular subscales in which significant effects may have occurred. Compared to the conventional-treatment group, the folic acid treatment group demonstrated significant increases in the Full Scale IQ (P = 0.028, effect size *d* = 0.153), Information (P = 0.031, effect size *d* = 0.157), and Digit Span (P = 0.009, effect size *d* = 0.172) scores from baseline to the twelfth month. In addition to the intervention effects, baseline Hcy concentrations had an important association with cognitive performance over time. Elevated Hcy concentrations at baseline were associated with poorer cognitive performance on the Full Scale IQ (estimate value = −0.118, P = 0.029), Information (estimate value = −0.078, P = 0.000), and Digit Span (estimate value = −0.053, P = 0.006) tests at month twelve (Table 5).

## Discussion

In this population-based randomized controlled trial, the administration of a daily oral folic acid supplement (400 μg) to individuals with MCI for twelve months was associated with significant improvements in global cognitive function compared to individuals in the conventional-treatment group, particularly in memory tasks. Additionally, we observed a significant reduction in the levels of peripheral inflammatory cytokines including IL-6, TNF-α, circulating Aβ-42, and plasma Hcy concentrations, in the treatment group during the follow-up period.

### Association between peripheral inflammatory cytokine levels and cognitive impairment/dementia

Peripheral inflammatory cytokines have emerged as a potential class of cytokines that may be useful for predicting individuals who are at a greater risk of developing dementia. Several population-based studies have reported an association between peripheral inflammatory cytokine levels and the risk of cognitive impairment/dementia^24^,^25^. Peripheral inflammatory cytokines, particularly IL-6, TNF-α, and IL-1β^26^,^27^, readily cross the blood–brain barrier (BBB) via saturable systems, suggesting that peripheral measures do reflect direct changes in the brain^28^. Therefore, peripheral inflammation is an important and potentially modifiable condition for patients who complain of changes in cognitive performance.

Dementia develops over a long preclinical period, and its association with inflammatory markers may be a consequence of the disease process rather than a causal association. Therefore, investigations of the role of inflammatory markers in the early stages of the disease process where cognitive decline is apparent but has not yet developed into the full clinical dementia syndrome are important. In this context, we selected individuals suffering from MCI and utilized a folic acid intervention to attenuate inflammation, as we believe this treatment would be a feasible public health strategy for preventing dementia.

### Effect of folic acid supplementation on peripheral inflammatory cytokine levels

Folate is a cofactor in one-carbon metabolism, during which it promotes the remethylation of homocysteine – a cytotoxic sulfur-containing amino acid that links the methionine cycle with the folate cycle^29^,^30^. In this trial, daily folic acid supplementation reduced the plasma Hcy concentrations in elderly individuals with MCI.

Hcy is an independent risk factor for cognitive impairment and AD^31^,^32^. However, convincing evidence supporting a causal relationship between elevated Hcy concentrations and the risk of AD is lacking. Inflammation is one of the earliest neuropathological events in AD. Hcy is associated with a proinflammatory response in *in vitro* studies, animal models, and humans^32^,^33^. Elevated concentrations of Hcy and low concentrations of folate may exert detrimental effects on cognitive function by inducing systemic inflammation^34^,^35^. Furthermore, folate plays a well-known role in maintaining the immune system^36^. Here, reducing the Hcy concentrations via folic acid supplementation ameliorated these effects.

In our trial, the serum IL-6, TNF-α and Aβ-42 percentages showed substantial decreases in both groups over the twelve-month period in the repeated-measures ANOVAs. However, this decrease was greater in the intervention group than in the conventional-treatment group. Thus, the marked reduction in the Hcy concentrations by the twelve-month folic acid supplementation (400 μg/day) effectively reduced the levels of peripheral inflammatory cytokines, with subsequent positive effects on cognition in elderly individuals with MCI.

### Folic acid supplementation significantly improved cognitive function by regulating peripheral inflammatory cytokines

In this RCT, daily oral administration of a 400-μg folic acid supplement to patients with MCI for 12 months can significantly improve cognitive performance, which is similar to the recent results from the FACIT trial showing that an daily oral administration of a 800-μg folic acid supplement for 36 months improves performance on tests that measure information processing speed and memory, domains that are known to decline with age^37^. In comparison, the FACIT trial was conducted in older adults with higher total homocysteine (tHcy) concentrations, and our trial was repeated in populations with MCI. Based on the relative doses of folic acid used and the short follow-up time (12 months), we obtained similar conclusions. RCTs of a tHcy-lowering treatment using B-vitamin supplementation have shown inconsistent results for cognitive function^38^,^39^,^40^. The meta-analysis by Clarke *et al*.^40^ concluded that B-vitamins do not influence cognitive aging in older people. The characteristics of the observed populations are different between our study and that of Clarke. The population of our study consists of older Chinese subjects. Their serum vitamin B_12_ concentrations are normal, but their serum folate levels are lower and homocysteine levels are higher. Methodological limitations of individual studies cannot be assumed to have been neutralized by the simple expedient of pooling on this scale. Factors such as dosage, vitamin combination, duration of treatment and the population treated may account for some of the discrepancies^38^,^39^.

In this trial, folic acid supplementation beneficially affected global cognitive function, specifically participants’ performance on the Information and Digit Span tasks. The Information test is a valid indicator of long-term memory, whereas the Digit Span test examines attention/short-term memory^41^,^42^. Participants allocated to the folic acid supplementation group performed significantly better on the Information and Digit Span subtests than individuals allocated to the conventional-treatment group. Poor performance on memory tests is a main characteristic of the cognitive deficits in patients with MCI. Furthermore, memory decline has been associated with hippocampal lesions^43^ and AD^44^. The hippocampus is located in the temporal lobe and plays important roles in the consolidation of information from short-term to long-term memory. Deterioration of the hippocampus precedes and leads to memory impairment in late adulthood^45^,^46^. In a recent clinical trial of individuals with mild cognitive impairment, treatment with B-vitamins appears to slow cognitive decline^37^, total brain atrophy^47^, and regional brain atrophy^48^. Clinical trials to test this hypothesis are warranted.

Vitamin B_12_ and folate are essential factors for the remethylation of homocysteine to methionine and the subsequent production of S-adenosyl methionine. Folate and vitamin B_12_ deficiencies have been associated with neurodegenerative disease^49^. An interaction between folate intake and vitamin B_12_ intake was observed such that less marked cognitive decline was observed in individuals who also took high-dose vitamin B_12_–containing supplements^50^. In subjects with normal vitamin B_12_ status, high serum folate levels (59 nmol/L) were associated with protection from cognitive impairment. However, the relation between high serum folate levels and cognitive impairment was reversed in subjects who had a low vitamin B_12_ status^51^. In our study, daily oral administration of a 400-μg folic acid supplement to individuals with MCI and a high vitamin B_12_ status (>569.24 pg/mL) for 12 months is more likely to improve cognitive performance on the Full Scale IQ test. There were no significant findings in the group with the low vitamin B_12_ status. We also analyzed the interaction between the subjects’ vitamin B_12_ status and serum folate levels using the cut-off for absolute vitamin B_12_ deficiency (148 pmol/L (200 pg/mL))^52^. However, only 3 subjects had a vitamin B_12_ deficiency and were further grouped by the intervention – 2 subjects in the intervention group and 1 subject in the control group. The sample size was too small. Therefore, we did not perform an in-depth analysis. We hope these findings can offer a reference for a similar trial.

Low-grade peripheral inflammation may contribute to the hippocampal changes that underlie these deficits. For instance, low-grade peripheral inflammation is associated with increased cognitive decline, including reduced hippocampal volume, which, as mentioned above, is a structure critical for memory formation and is associated with dementia^53^. Additionally, the hippocampus has the highest levels of receptors for the inflammatory cytokines IL-6 and IL-1β in the brain^54^. Cytokine receptors are abundantly expressed in the hippocampus, and increased peripheral cytokine levels have been associated with disrupted hippocampal stem cell function, reduced hippocampal volume, and reduced memory performance^55^,^56^,^57^. Therefore, not surprisingly, many studies have linked increases in low-grade peripheral inflammation to cognitive impairment, and have specifically linked memory tasks to hippocampal deterioration.

Folate may selectively benefit the hippocampus because the hippocampus is one of the unique regions in the brain that undergoes cell renewal and DNA replication; therefore, it may have a greater dependence on vitamins that are essential for nucleotide synthesis^58^. In our trial, all cognitive measures were converted into Z scores based on the pooled MCI group because the cognitive effects of folic acid supplementation might vary by cognitive domain. After converting the crude cognitive test scores into Z scores, folic acid supplementation significantly improved the participants’ cognitive performance on memory tests and decreased the levels of peripheral inflammatory cytokines, as shown in the repeated-measures ANOVAs. Over the course of the intervention, significant improvements in the Full Scale IQ, Information, and Digit Span tests were negatively associated with the levels of peripheral inflammatory cytokines in elderly subjects with MCI in the mixed-model, repeated-measures analysis. Based on the available evidence folic acid supplementation among participants with MCI improves cognitive performance in the short-term and long-term memory domains by reducing the peripheral inflammatory cytokine levels.

### Limitations and strengths

Several elements of our study support the veracity of the findings. First, we used a standard measure of global cognitive function. Several important, aging-sensitive cognitive functions were studied, and the level of cognitive function over time was used as an outcome measure. Moreover, the trial had sufficient power to detect a clinically meaningful difference in cognitive function between the treatments. Second, to our knowledge, cytokines have typically been assessed (e.g., IL-1β, IL-6, and TNF-α) in patients with MCI/AD and compared to controls in observational studies, not clinical trials^59^,^60^. Thus, the scope of our assessment contributes to a broader understanding of the relations between select markers of immune function and MCI/AD in response to improvements in nutritional status with folic acid supplementation. Finally, compliance with supplementation use was high, as only 9% of participants failed to complete the full trial, and adverse effects were infrequent.

Although our results are promising, some limitations should be considered when interpreting our findings. First, the intervention period (twelve months) may not be long enough to reveal the full spectrum of the effects on cognitive improvement. Longer interventions with folic acid supplementation might lead to greater changes. Second, the optimal dosage of folic acid needed to reduce inflammation and improve cognitive function is unknown. Although the present study provides some insights, larger dosages may produce different effects than those observed in the current investigation. Third, if a community-based RCT is conducted in China, researchers must be responsible for the complete study (conceiving and designing the study, data acquisition, data analysis, etc.). The researchers must know all the details (for example, know whether test subjects are in the control group or the experimental group), and so the researchers themselves cannot be blinded. Therefore, our study used a single-blind experimental design. Individual subjects did not know whether they were so-called “test” subjects or members of the control group. The conclusions should be verified in a double-blinded trial in the future. Finally, the most frail and low-functioning individuals did not complete the trial. These individuals had lower scores on the cognitive tasks and lower folate levels compared to the individuals who completed the study. The use of the mixed-model, longitudinal data analysis technique has partially resolved the bias induced by the loss of these individuals; however, an underestimation of the identified associations is likely.

In conclusion, folic acid has anti-inflammatory and memory-enhancing properties. Supplementing the subjects’ diets with 400 μg of folic acid daily for twelve months significantly improved their cognitive performance and reduced systemic inflammation. Cognitive improvement resulting from folic acid supplementation depends on regulating the peripheral inflammatory cytokine levels. Folic acid is a promising treatment for individuals with MCI.

## Methods

### Study design and participants

This study is a single-center randomized controlled trial with an intent-to-treat paradigm that seeks to investigate the effects of a twelve-month folic acid supplement intervention on cognition and peripheral inflammatory cytokine levels in older adults with MCI. Participants were enrolled between March 2013 and April 2013, based on the following criteria: 1) age 65+; 2) absence of terminal illness or mental disorders (i.e., major depression, schizophrenia, bipolar disorder, etc.); 3) not using any nutritional supplementation known to interfere with nutrition status, folate metabolism, or cognitive function in the three months prior to recruitment; and 4) not living in a nursing home or being on a waiting list for a nursing home. Using random cluster sampling, six geographically convenient communities with a high proportion of older residents who were all native Chinese speakers were selected from the Binhai New District, Tianjin, China. Of the 4215 selected individuals, 2816 (66.8%) agreed to participate, but only 2317 individuals who met the inclusion criteria participated in the clinical, physical and neuropsychological examinations.

Two hundred ten subjects with MCI were selected using previously determined criteria for MCI (Fig. 1). Ninety-seven percent of participants in both the intervention and control groups were living in the community at recruitment, and the participants’ family doctors considered all patients suitable for the study. Of the 210 MCI individuals screened, 168 met the study criteria and were randomly assigned in the folic acid supplementation or control groups. Cognitive function was assessed at baseline and follow-ups at the sixth and twelfth months. Please see Fig. 1 for the CONSORT flow chart.

The study was conducted in compliance with the ethical principles of the Declaration of Helsinki and was approved by the medical ethics committee of Tianjin Medical University, China. Each subject provided written informed consent prior to study entry. This trial has been registered on May 4^th^ 2013 with trial number ChiCTR-TRC-13003227 (http://www.chictr.org.cn/showproj.aspx?proj=6332).

### Data collection

All participants completed a basic sociodemographic and medical history questionnaire and reported the list of medications they were taking at the baseline assessment. The interview included the following information: age (in years), sex, education (in years), marital status, occupation, whether they smoked (ever or never), the number of packs smoked per year, number of depressive symptoms, heart disease (self-reported history of myocardial infarction, digitalis use, or angina pectoris), hypertension (self-reported history, measured blood pressure ≥160 mmHg systolic or ≥95 mmHg diastolic, or use of antihypertensive medications), history of stroke (self-report), and diabetes mellitus (self-report or antidiabetic medication use).

### Assessment of cognitive function

The main outcome of the current study was cognitive function, which was measured using the Chinese version of the Wechsler Adult Intelligence Scale-Revised (WAIS-RC)^22^. The WAIS-RC includes 11 sub-tests: Information, Similarities, Vocabulary, Comprehension, Arithmetic, Digit Span, Block Design, Picture Completion, Digit Symbol-Coding, Object Assembly, and Picture Arrangement. The neuropsychological assessments were administered by trained physicians at the baseline assessment and at the six- and twelve-month time points during treatment. We used age-appropriate norms from the Chinese standardization to calculate the intelligence quotient (IQ) and index scores^61^.

### Definition of MCI

MCI was determined in accordance with the modified Petersen’s criteria^62^, as follows:

1. Subjective memory complaint with a duration of at least 2 weeks.
2. Based on the individual’s age and education, objective memory impairments was defined as performing at least 1.5 SD below age- and education-matched controls on the mini-mental state examination memory subtask^63^.
3. Normal general cognitive function impairment was defined as a test performance that was more than 1.5 SD below age- and education-specific norms.
4. Essentially preserved activities of daily living, as measured by the Activities of Daily Living scale (i.e., a score of <26)^64^.
5. Absence of dementia (Diagnostic and Statistical Manual of Mental Disorders-IV), AD (the National Institute of Neurological and Communicative Disorders and Stroke and the Alzheimer’s Disease and Related Disorders Association), psychiatric disorders, cerebral damage, or physical diseases that may account for cognitive impairment.

### Randomization and intervention

After baseline screening, eligible participants were randomly assigned to the folic acid supplementation group or the conventional-treatment group. The study sponsor generated the randomization sequence with a computer.

Eligible participants who were randomized to the folic acid supplementation group received one pill per day. Folic acid tablets were formulated as a daily oral dose of one tablet consisting of 400 μg of folic acid (Beijing Scrianen Pharmaceutical Co. Ltd., China; 400 μg/tablet; state medical permit No.: H10970079) for the entire twelve-month period. The other group only received the conventional treatment. At baseline, all MCI subjects lived in the same community. The subjects only received non-pharmacological interventions for preventing, reducing, or postponing cognitive decline in late life and dietary recommendations based on a booklet (Guides to Enhance Elderly Memory). The conventional treatment was described above. Adherence was encouraged and monitored in both groups throughout the trial by telephone interviews at 15 time points and by blood assay at the baseline, six- and twelve-month assessments.

Because there was no feasible way to blind the patients’ group allocations, it may not be feasible or ethical to provide a sham procedure to make blinding possible. However, minimizing measurement bias in this situation may be best accomplished by recruiting a trial investigator, outcome assessors, and data analysts in an attempt to decrease biased classification of the outcomes or unexpected side effects.

### Assessment of peripheral inflammatory cytokine and folate levels

Blood samples were aseptically collected at the baseline, six-, and twelve-month assessments by venipuncture after a 10-to-12-h overnight fast. The concentrations of folate and vitamin B_12_ were determined on the same day using the AbbottArchitect-i2000SR automated chemiluminescence immunoassay system and its supporting kit (Abbott, USA). Additionally, the serum Hcy concentrations were determined with a Hitachi 7180 automatic biochemistry analyzer (Japan) using the enzymatic conversion method. The kit was supplied by Beijing Strong Biotechnologies, Inc. (China). The serum levels of IL-6, TNF-α, Aβ-40, and Aβ-42 were measured with a high-sensitivity sandwich ELISA kit (R&D Systems Inc., Minneapolis, MN, USA). All measurements were performed in duplicate, and the average values were used in the statistical analyses.

### Statistical analysis

Intention-to-treat (ITT) analyses were performed using the last observation carried forward method for subjects who were lost to follow-up or had missing data. Baseline characteristics of the intervention and control groups were compared using Pearson’s chi-square tests or Fisher’s exact test for categorical variables and the *t* test or nonparametric Wilcoxon ranked sum test for continuous variables; post hoc comparisons were performed using the Bonferroni test for multiple comparisons. Because the cognitive effects of folic acid supplementation might vary by cognitive domain, domain-based Z scores were determined before and after treatment. Crude cognitive test scores at baseline and after 6 months (or 12 months) were pooled to calculate the grand mean and SD per test. Repeated-measures ANOVAs were used to evaluate the effects of the folic acid intervention and conventional treatment on cognitive performance and on the levels of peripheral inflammatory cytokines over the 12-month treatment period. For easier comparisons, an effect size for each test was calculated by dividing the difference between the treatments by the standard deviation of the test result for the entire sample at baseline. Mixed-model repeated-measures ANOVAs were used to evaluate hypotheses concerning differences in the changes observed between the folic acid supplementation group and the control group. Models were developed for each of the cognitive outcome variables. The critical test of the effectiveness was the presence of an effect of the folic acid supplementation compared to the control treatment over time (i.e., folic acid supplementation improved cognitive functioning over time). Mixed models yield an intention-to-treat analysis by using all available measurement points for each participant under the assumption that withdrawal data are missing at random. We attempted to preserve the benefits of randomization, which is intended to ensure that differences in outcomes observed between groups are solely the result of the treatment, thus reducing the risk of selection bias. All analyses were performed using SPSS PASW Statistics for Windows, version 18.0 (SPSS Inc. Released 2009,: Chicago, IL, USA).

## Additional Information

**How to cite this article**: Ma, F. *et al*. Folic acid supplementation improves cognitive function by reducing the levels of peripheral inflammatory cytokines in elderly Chinese subjects with MCI. *Sci. Rep.* **6**, 37486; doi: 10.1038/srep37486 (2016).

**Publisher's note:** Springer Nature remains neutral with regard to jurisdictional claims in published maps and institutional affiliations.

## Figures and Tables

**Table 1:** Baseline characteristics of the study population.

Profile	Folic acid group (n = 84)	Control group (n = 84)	P-value^a^
Demographics			
Age at screening (years)	73.71 ± 2.57	73.52 ± 3.03	0.77
Female	57 (67.86)	58 (69.05)	0.43
Total education (years)	9.14 ± 3.53	9.81 ± 3.09	0.15
Lifestyle			
Smoker—ever	51 (60.71)	53 (63.10)	0.19
Alcohol (units/week)	9.09 ± 9.11	8.51 ± 8.33	0.13
Systolic BP (mmHg)	145 ± 22	147 ± 24	0.66
Diastolic BP (mmHg)	82 ± 11	81 ± 10	0.54
BMI (kg/m^2^)	24.53 ± 3.79	25.01 ± 4.11	0.17
Medical history			
Diabetes mellitus—ever	5 (5.95)	4 (4.76)	0.13
Hypertension	6 (7.14)	7 (8.33)	0.64
Vascular disease	11 (13.10)	10 (11.90)	0.24
MMSE	25.60 ± 2.22	26.13 ± 2.51	0.33
Folate (ng/mL)	7.01 ± 1.01	6.33 ± 0.97	0.44
Hcy (mmol/L)	13.65 ± 4.82	12.19 ± 3.85	0.57
Vitamin B_12_(pg/mL)	571.25 ± 23.37	568.48 ± 16.33	0.46

Results are shown as n (%) for the chi-square or Fisher’s exact tests and as the mean ± SD for independent *t*-tests (two-tailed). ^a^P < 0.01: significant difference between groups. Abbreviations: BP, blood pressure; BMI, Body Mass Index; Hcy, homocysteine.

**Table 2:** The levels of serum biomarker parameters in the folic acid and control groups at baseline, 6 months, and 12 months.

Item	Groups	Cases (n)	Treatment time*			Repeated measures^§^		
			Before treatment	6 months	12 months	Interaction effect, P (*ηp*^*2*^)	Time effect, P (*ηp*^*2*^)	Group effect, P (*ηp*^*2*^)
Folate (ng/mL)	Intervention	84	7.01 ± 3.64	8.97 ± 0.62	9.44 ± 0.33	0.011 (0.703)	0.016 (0.117)	0.229 (0.041)
	Control	84	5.79 ± 2.67	5.96 ± 2.11	5.88 ± 2.03			
Hcy (μmol/L)	Intervention	84	13.65 ± 4.82	9.49 ± 5.72	8.21 ± 4.22	0.009 (0.644)	0.046 (0.098)	0.723 (0.007)
	Control	84	12.19 ± 3.85	10.97 ± 2.42	11.33 ± 4.51			
Vitamin B_12_(pg/mL)	Intervention	84	571.25 ± 23.37	583.78 ± 25.44	571.33 ± 20.45	0.279 (0.103)	0.588 (0.094)	0.271 (0.211)
	Control	84	568.48 ± 16.33	537.43 ± 27.39	555.45 ± 19.58			
Aβ-40(pg/mL)	Intervention	84	53.06 ± 1.02	52.44 ± 1.28	51.50 ± 1.08	0.306 (0.042)	0.677 (0.016)	0.479 (0.013)
	Control	84	53.55 ± 1.84	53.45 ± 1.27	52.49 ± 1.07			
Aβ-42(pg/mL)	Intervention	84	53.07 ± 1.04	50.23 ± 1.29	49.16 ± 1.09	0.013 (0.687)	0.609 (0.028)	0.463 (0.021)
	Control	84	53.55 ± 1.84	53.45 ± 1.27	52.49 ± 1.07			
IL-6(pg/mL)	Intervention	84	9.48 ± 0.22	9.21 ± 0.39	7.71 ± 0.21	0.025 (0.477)	0.247 (0.047)	0.948 (0.001)
	Control	84	9.56 ± 0.19	9.39 ± 0.28	9.01 ± 0.17			
TNF-α(pg/mL)	Intervention	84	138.41 ± 2.44	126.21 ± 7.97	101.09 ± 6.76	0.009(0.709)	0.011*(0.123)	0.229 (0.044)
	Control	84	139.21 ± 2.73	140.21 ± 6.58	138.77 ± 6.97			

^§^ηp^2^ describes the percentage of variance in the dependent variable explained by a predictor variable. P values for each group (intervention versus control) were derived from the ANCOVA and adjusted for the respective baseline values.

Abbreviations: Hcy, homocysteine; IL-6, interleukin-6; TNF-α, tumor necrosis factor-α.

**Table 3:** Neuropsychological test results at baseline, 6 months, and 12 months.

Cognitive test	Groups	Cases (n)	Treatment time			Repeated measures§		
			Before treatment	6 months	12 months	Interaction effect, P (ηp^2^)	Time effect, P (ηp^2^)	Group effect, P (ηp^2^)
Full Scale IQ	Intervention	84	−1.10 ± 0.95	0.25 ± 0.09	0.85 ± 1.04	0.044 (0.082)	0.286 (0.049)	0.376 (0.022)
	Control	84	−0.87 ± 0.90	0.11 ± 0.18	0.16 ± 0.61			
Information	Intervention	84	−0.61 ± 0.24	0.54 ± 0.86	1.15 ± 1.10	0.000 (0.452)	0.609 (0.021)	0.507 (0.023)
	Control	84	−0.70 ± 0.04	0.23 ± 1.02	0.25 ± 0.98			
Comprehension	Intervention	84	1.05 ± 1.08	1. 01 ± 0.88	0.94 ± 0.20	0.059 (0.087)	0.116 (0.082)	0.359 (0.034)
	Control	84	1.09 ± 1.14	1.08 ± 0.40	1.21 ± 0.73			
Digit Span	Intervention	84	1.37 ± 0.21	1.77 ± 1.09	1.96 ± 0.88	0.000 (0.388)	0.063 (0.092)	0.509 (0.016)
	Control	84	1.44 ± 0.52	1.54 ± 0.63	1.39 ± 1.15			
Vocabulary	Intervention	84	−1.05 ± 0.15	0.14 ± 1.07	0.12 ± 0.92	0.189 (0.055)	0.144 (0.064)	0.854 (0.007)
	Control	84	−1.07 ± 0.37	0. 1 ± 1.13	0.16 ± 0.76			
Arithmetic	Intervention	84	−1.00 ± 0.58	−1.00 ± 1.15	−0.91 ± 0.58	0.388 (0.032)	0.679 (0.015)	0.471 (0.014)
	Control	84	−0.76 ± 1.12	−1.13 ± 0.32	−0.37 ± 0.80			
Similarities	Intervention	84	−1.04 ± 0.82	1.02 ± 1.12	0.98 ± 0.30	0.688 (0.015)	0.706 (0.015)	0.516 (0.014)
	Control	84	−1.05 ± 1.09	1.11 ± 0.22	0.94 ± 0.87			
Picture Completion	Intervention	84	−0.31 ± 0.55	0.81 ± 0.60	0.72 ± 1.15	0.282 (0.087)	0.756 (0.012)	0.842 (0.009)
	Control	84	−0.87 ± 0.81	1.09 ± 0.31	0.83 ± 1.12			
Picture Arrangement	Intervention	84	−1.05 ± 1.15	0.94 ± 0.62	0.11 ± 0.54	0.116 (0.096)	0.225 (0.0523)	0.839 (0.007)
	Control	84	−0.94 ± 0.25	−1.05 ± 0.85	0.10 ± 1.10			
Block Design	Intervention	84	−1.02 ± 1.05	−0.99 ± 0.94	1.01 ± 0.11	0.385 (0.064)	0.242 (0.059)	0.309 (0.033)
	Control	84	−1.23 ± 0.75	−0.71 ± 0.39	0.98 ± 1.14			
Object Assembly	Intervention	84	1.53 ± 0.84	−1.15 ± 0.27	0.62 ± 1.11	0.721 (0.012)	0.260 (0.046)	0.657 (0.022)
	Control	84	1.15 ± 1.13	−0.63 ± 0.36	0.53 ± 0.77			
Digit Symbol	Intervention	84	−0.87 ± 1.15	−0.23 ± 0.51	1.09 ± 0.64	0.911 (0.007)	0.255 (0.052)	0.751 (0.012)
	Control	84	−1.15 ± 0.78	−0.52 ± 0.35	0.63 ± 1.13			

Z scores are shown; the data are standardized according to the crude cognitive test scores at baseline and after 6 months (or 12 months), which were pooled to calculate the grand mean and SD per test.

^§^ηp^2^ describes the percentage of variance in the dependent variable explained by a predictor variable. P values for each group (intervention versus control) were derived from the ANCOVA and adjusted for the respective baseline values.

P values for each group (intervention versus control) were derived from the ANCOVA and adjusted for the respective baseline values. Abbreviations: IQ, Intelligence Quotient.

**Table 4:** Interaction between vitamin B_12_ status and serum folate levels in relation to the Full Scale IQ at baseline, 6 months and 12 months.

Vitamin B_12_ status	Groups	Cases (n)	Treatment time			Repeated measures§		
			Before treatment	6 months	12 months	Interaction effect P (ηp^2^)	Time effect P (ηp^2^)	Group effect P (ηp^2^)
High vitamin B_12_ status (>569.24 pg/mL)	Intervention	32	−1.11 ± 0.92	0.24 ± 0.07	0.82 ± 1.07	0.032 (0.109)	0.279 (0.043)	0.158 (0.671)
	Control	30	−0.86 ± 0.91	0.12 ± 0.15	0.13 ± 0.63			
Low vitamin B_12_ status (<569.24 pg/mL)	Intervention	52	−1.02 ± 0.54	−1.07 ± 1.05	−0.92 ± 0.52	0.351 (0.019)	0.552 (0.008)	0.477 (0.123)
	Control	54	−0.72 ± 1.02	−1.10 ± 0.33	−0.33 ± 0.61			

According to the crude cognitive test scores at baseline and after 6 months (or 12 months), which were pooled to calculate the grand mean and SD per test.

^§^ηp^2^ describes the percentage of variance in the dependent variable explained by a predictor variable. P values for each group (intervention versus control) were derived from the ANCOVA and adjusted for the respective baseline values.

P values for each group (intervention versus control) were derived from the ANCOVA and adjusted for the respective baseline values. Abbreviations: IQ, Intelligence Quotient.

**Table 5:** Mixed-model analysis describing the association between the Full Scale IQ, Information, and Digit Span test scores and the laboratory variables at baseline.

Cognition score	Estimate	SEMs	*t* test	P value	95% CI		Effect size, P
					Lower	Upper	
Full Scale IQ							
Intercept	20.561	0.258	15.471	0.009	19.433	24.294	
Hcy (baseline)	−0.118	0.034	−2.073	0.029	−0.230	−0.016	
IL-6 (baseline)	−0.154	0.021	−2.051	0.037	−0.262	−0.051	
TNF-α(baseline)	−0.583	0.197	−3.251	0.013	−0.726	−0.458	
Aβ-42 (baseline)	−0.297	0.264	−1.148	0.267	−0.595	0.016	
FA × wave							0.153^b^, 0.028
FA × Baseline^a^	0.001	0.001					
FA × 6 months	−0.238	0.225	−1.093	0.188	−0.749	0.224	
FA × 12 months	0.512	0.243	2.503	0.027	0.074	1.145	
Information							
Intercept	6.163	0.231	18.466	0.007	5.638	6.702	
Hcy (baseline)	−0.078	0.029	−4.873	0.000	−0.114	−0.045	
IL-6 (baseline)	−0.141	0.015	−4.401	0.0123	−0.246	−0.035	
TNF-α(baseline)	−0.534	0.029	−4.144	0.015	−0.685	−0.409	
Aβ-42 (baseline)	−0.071	0.163	−1.021	0.343	−0.246	0.118	
FA × wave							0.157^b^, 0.031
FA × Baseline^a^	0.001	0.001					
FA × 6 months	−0.068	0.109	−0.567	0.573	−0.303	0.169	
FA × 12 months	0.222	0.133	2.040	0.026	0.013	0.462	
Digit span							
Intercept	4.727	0.257	15.977	0.001	4.152	5.303	
Hcy (baseline)	−0.053	0.032	−3.277	0.006	−0.087	−0.032	
IL-6 (baseline)	−0.142	0.016	−4.403	0.011	−0.249	−0.040	
TNF-α(baseline)	−0.587	0.069	−4.159	0.012	−0.732	−0.461	
Aβ-42 (baseline)	−0.159	0.157	−1.629	0.113	−0.372	0.048	
FA × wave							0.172^b^, 0.009
FA × Baseline^a^	0.001	0.001					
FA × 6months	0.030	0.129	0.245	0.827	−0.211	0.259	
FA × 12 months	0.309	0.148	2.571	0.007	0.094	0.588	

Abbreviations: IQ, Intelligence Quotient; Hcy, homocysteine; IL-6, Interleukin-6; TNF-α, tumor necrosis factor-α; FA, folate; Aβ, amyloid β-protein.

^a^Reference category.

^b^Cohen’s d refers to the magnitude of the standardized mean effect (i.e., the mean difference between two groups in SD units). Significance was set at P < 0.05.
